# Carbonic anhydrase IX induction defines a heterogeneous cancer cell response to hypoxia and mediates stem cell-like properties and sensitivity to HDAC inhibition

**DOI:** 10.18632/oncotarget.4989

**Published:** 2015-07-22

**Authors:** Ioanna Ledaki, Alan McIntyre, Simon Wigfield, Francesca Buffa, Simon McGowan, Dilair Baban, Ji-liang Li, Adrian L. Harris

**Affiliations:** ^1^ Molecular Oncology Laboratories, Department of Oncology, University of Oxford, Weatherall Institute of Molecular Medicine, Oxford, UK; ^2^ High Throughput Genomics, Wellcome Trust Centre for Human Genetics, University of Oxford, Oxford, UK

**Keywords:** carbonic anhydrase IX, stem cells, hypoxia, EMT, tumour heterogeneity

## Abstract

Carbonic anhydrase IX (CAIX) is strongly induced by hypoxia and its overexpression is associated with poor therapeutic outcome in cancer. Here, we report that hypoxia promotes tumour heterogeneity through the epigenetic regulation of CAIX. Based on hypoxic CAIX expression we identify and characterize two distinct populations of tumour cells, one that has inducible expression of CAIX and one that does not. The CAIX+ve population is enriched with cells expressing cancer stem cell markers and which have high self-renewal capacity. We show that differential CAIX expression is due to differences in chromatin structure. To further investigate the relationship between chromatin organization and hypoxic induction of CAIX expression we investigated the effect of JQ1 an inhibitor of BET bromodomain proteins and A366 a selective inhibitor of the H3K9 methyltransferase G9a/GLP. We identified that these drugs were able to modulate hypoxic CAIX expression induction. This further highlights the role of epigenetic modification in adaption to hypoxia and also in regulation of heterogeneity of cells within tumours. Interestingly, we identified that the two subpopulations show a differential sensitivity to HDAC inhibitors, NaBu or SAHA, with the CAIX positive showing greater sensitivity to treatment. We propose that drugs modulating chromatin regulation of expression may be used to reduce heterogeneity induced by hypoxia and could in combination have significant clinical consequences.

## INTRODUCTION

During tumour development, rapid expansion and growth of cancer cells creates poorly vascularized regions, characterized by hypoxia, with irregular blood flow, low pH and nutrient starvation conditions which select for a more aggressive tumour phenotype [1].

Cellular responses and adaptation to hypoxia are mediated partly through stabilization of hypoxia inducible transcription factors (HIFs). The HIFs induce the transcription of many hypoxia-response genes, involved in angiogenesis e.g. vascular endothelial growth factor (*VEGF*), pH regulation e.g. carbonic anhydrase 9 (*CA9*), and alterations in cellular metabolism e.g. lactate dehydrogenase-A (*LDHA*) by binding to hypoxia-response elements (HRE) in their promoters [2]. CAIX catalyzes the reversible hydration of CO_2_ to bicarbonate and a proton. This enables tumours to maintain a more neutral intracellular pH (pH_i_) which promotes survival but produces a more acidic extracellular pH (pH_e_) [3] which promotes invasion and metastasis. The expression of CAIX is associated with poor survival in most cancers, [4, 5, 6].

Cancer stem cells (CSC) generate cancer cell heterogeneity commonly observed in clinical samples. However, most treatment strategies fail to address tumour heterogeneity sufficiently. Hypoxia is linked with stem cell niches. For example, the transcription factors HIF1α and HIF2α have been linked to the stimulation of cancer stem cells (CSCs) in glioblastoma [7, 8]. Wicha *et al* showed that hypoxia induced by inhibition of angiogenesis increases the population of breast CSCs in xenografts [9]. Accordingly, the poor patient survival and therapeutic resistance that is associated with hypoxia may be a result of increased proportion of CSCs in tumours [10]. Therefore, combination studies of currently identified stem cell markers with hypoxic markers may give a direct insight into the relationship between tumour heterogeneity in response to hypoxia and the role of stem cells.

A promising hypoxic marker for such studies is CAIX since its expression in normal tissues is limited to niches that correspond to sites harboring adult stem cells [11]. In addition, CAIX expression is required for the maintenance of CSC, and plays a role in the invasive potential of breast cancer cells and production of mammospheres [12, 13].

In this study we demonstrate that there is marked heterogeneity in CAIX expression within cancer cell lines, in contrast to many other HIF-target genes. The subpopulation that selectively induces CAIX is associated with the regulation of stemness. Our results provide further support to the notion that hypoxic regions serve as stem cell niches, with CAIX being a key stem cell regulator. The intracellular heterogeneity can be suppressed by inhibitors of a “reader” and a “modifier” of chromatin. We propose that strategies targeting the hypoxic subpopulations using inhibitors of chromatin regulation will help to develop new combination therapies against hypoxia and CAIX.

## RESULTS

### Differential expression of CAIX under hypoxia

**Figure 1:**
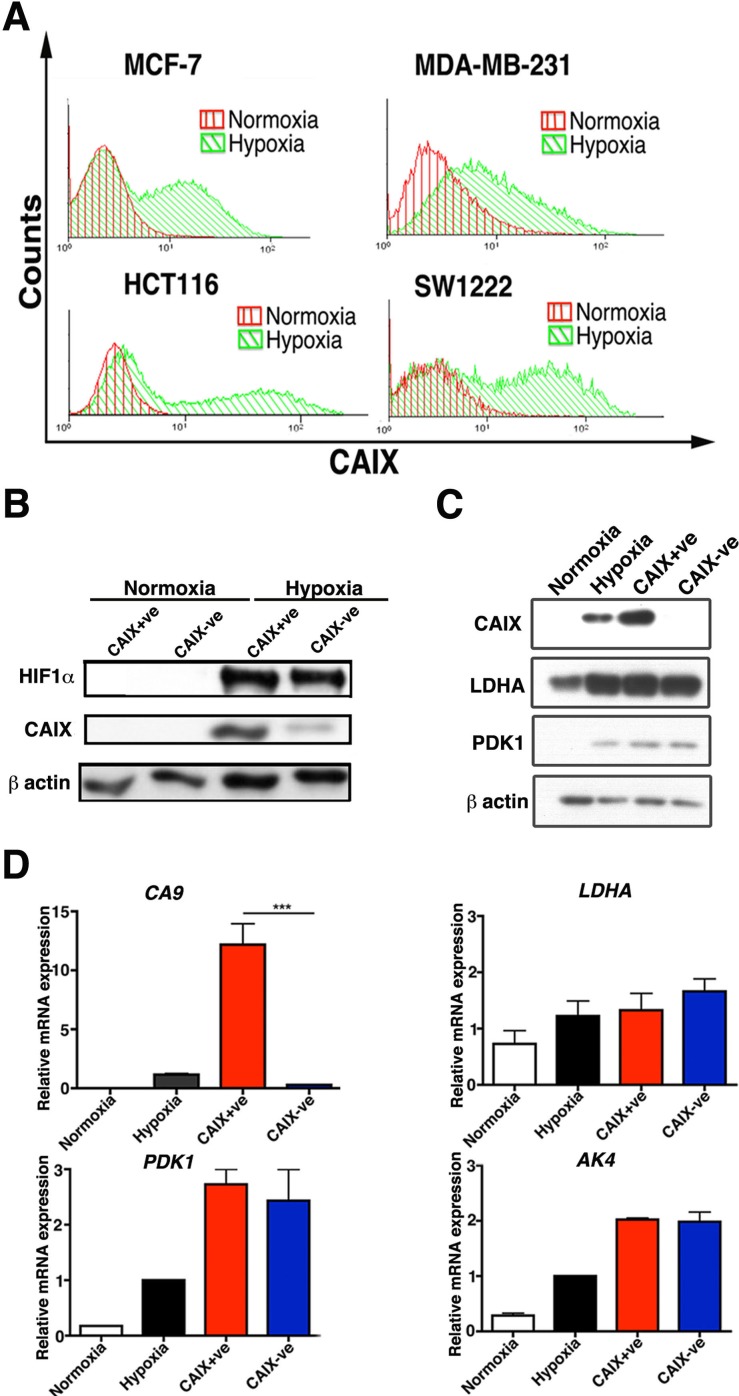
Hypoxic subpopulations of CAIX expression Two distinct subpopulations of CAIX-ve and CAIX+ve cells in MCF-7, MDA-MB-231, HCT116 and SW1222 lines stained with CAIX antibody and analysed by FACS **A.**. CAIX+ve and CAIX-ve sorted MCF-7 cells have the same protein levels of expression of HIF-1α, LDHA, PDK1 **B.** and **C.** and the same RNA levels of expression of HIF-1α, LDHA, PDK1 and AK4 **D.**. Error bars represent the mean ± SD. ***p < 0.001, *n* = 3.

To determine the induction profile of CAIX we performed a 72 hour time course under 0.1% O_2_. The maximum expression of CAIX was at 72 hours both at RNA (Figure S1A) and protein level (Figure S1B and Figure S2). Therefore, subsequent hypoxic (0.1% O_2_) experiments were performed at 72 hours. FACS analysis in four cell lines, MCF-7, HCT116, SW1222 and MDA-MD-231 showed that under hypoxic conditions (72 hours, 0.1% O_2_) there were two populations, in the first 3 cell lines, which differentially expressed CAIX (Figure 1A). The percentage of CAIX positive cells ranged from 30% to 50% in the four cell lines tested (Figure S3A and S3B). Using FACS sorting both CAIX positive (CAIX+ve) and CAIX negative (CAIX-ve) cells were isolated from the hypoxia incubated MCF-7 cell line, and CAIX expression was confirmed by Western blotting (Figure 1C). Importantly, HIF1α was analysed after the sorted populations were allowed to recover from hypoxia for 1 week, then re-exposed to 0.1% O_2_ for 24 hours. Both populations produced the same levels of HIF1α but CAIX remained differentially expressed (Figure 1B). Moreover, under hypoxia in both populations only CAIX had differential expression compared to the well-validated hypoxia regulated genes LDHA, pyruvate dehydrogenase kinase 1 (PDK1) and adenylate kinase 4 (AK4) (Figure 1B, 1C).

### Only the CAIX+ve population of the MCF-7 sorted cells has the ability to recapitulate the original expression pattern

**Figure 2:**
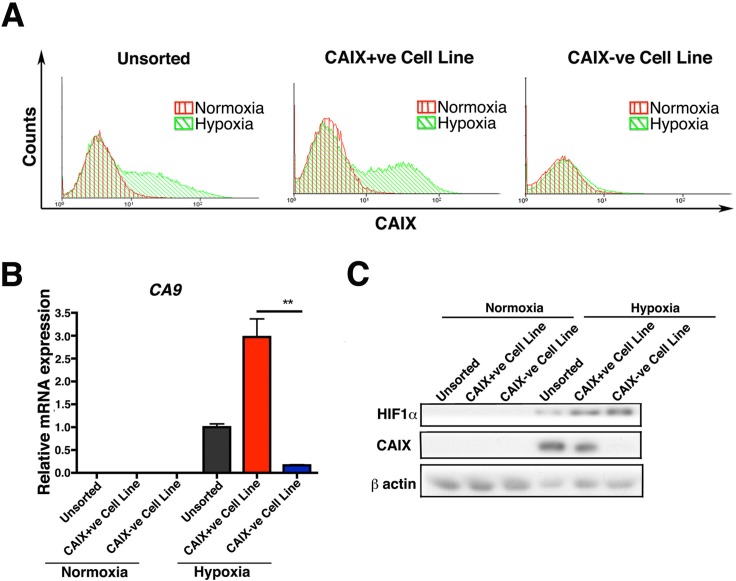
Long-term culture of CAIX+ve and CAIX-ve cells FACS analysis of CAIX shows that only the CAIX+ve population has the ability to recapitulate the original expression pattern and to reform both populations in long term (6 months) culture **A.**. The expression levels of CAIX in the long term (6 months) cultures of the sorted cells was confirmed both at RNA **B.** and protein level **C.**. Error bars represent the mean ± SD. ** = *p* < 0.01, *n* = 3.

We then performed two rounds of sorting of hypoxia-induced MCF-7 cells into CAIX+ve and CAIX-ve populations. After 3 weeks in culture both cell populations were reexamined for CAIX expression using FACS analysis. The CAIX+ve population had recapitulated the original unsorted phenotype, generating both populations of CAIX+ve and CAIX-ve cells. The CAIX-ve cells were not capable of reforming both populations and they remained mainly negative for CAIX, showing only a very low levels of CAIX expression (Figure S4A). In order to further enrich for CAIX+ve cells and CAIX-ve cells, a second round of FACS sorting was performed. The extreme high 10% of CAIX positive cells and the extreme low 10% of CAIX negative cells were sorted and cultured in normoxic conditions for six months. During long-term culture (6 months), the CAIX+ve cells became heterogeneous, containing both CAIX+ve and CAIX-ve populations when exposed to hypoxia, and thus recapitulated the original unsorted expression pattern, whereas the CAIX-ve cells continued to be homogenous, and negative for CAIX expression after exposure of the cells to 0.1% O_2_ hypoxia (Figure 2A). Thus we generated CAIX+ve and CAIX-ve cell lines. We verified the CAIX expression status in these at both, mRNA and protein levels (Figure 2B, 2C).

### Molecular signature of the MCF-7 CAIX positive and negative populations

**Figure 3:**
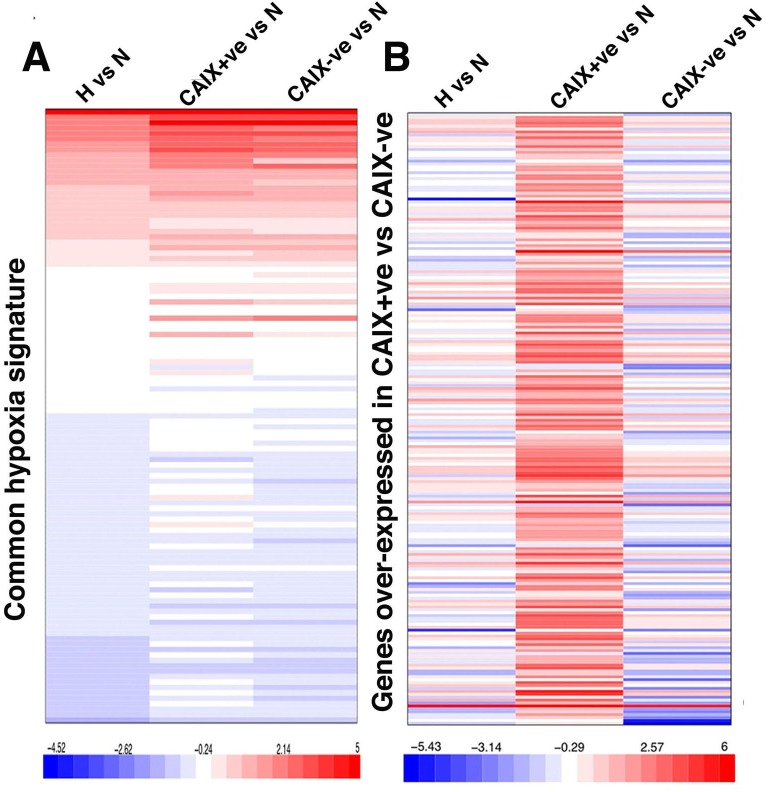
CAIX-positive and negative populations show similar hypoxia transcriptional response **A.** Expression of known hypoxia regulated genes, **B.** genes over-expressed in the CAIX positive population are shown in the three experimental conditions: 1) whole-population (left column), 2) CAIX+ve population (middle column) and 3) CAIX-ve population (right column) exposed to hypoxia normalized to the normoxia whole-population control. Bar legend shows the log2 count ratio between hypoxia and the normoxia.

In order to identify genes and pathways differentially expressed between CAIX+ve and CAIX-ve cells, RNA sequencing was performed. Cells were treated with hypoxia (0.1% O_2_) for 72 hours then stained for CAIX expression followed by cell sorting and RNA extraction. 2093 genes showed a greater than 2 fold up-regulation in the CAIX-ve population and 2670 showed a greater than 2 fold up-regulation in the CAIX+ve population. Comparing the gene expression pattern of these two populations under hypoxia, and the unsorted population under hypoxia with a breast cancer hypoxia expression signature [14] showed a similar core hypoxia response, with no difference in expression of well established hypoxia genes (Figure 3A). Interestingly, CAIX was the only hypoxia inducible gene distinguishing these two populations. Furthermore, the non-hypoxia regulated expression pattern of the CAIX+ve population was significantly different from both the CAIX-ve and unsorted cells (Figure 3B). Strikingly, KEGG pathway analysis showed that the differential CAIX+ve signature was significantly enriched for pathways involved in stem cell maintenance, such as the ABC transporters, Wnt and Hedgehog signaling, tumour invasion and metastasis (Table 1). In contrast, the CAIX-ve signature was enriched for cytokine-cytokine receptor interaction, MAPK signaling pathway, osteoblast differentiation and ECM-receptor interaction pathway (Table 2).

**Table 1:** KEGG analysis for CAIX+ve cells

Genes	Hyp*	Annotations
10	0.0009	ABC transporters
8	0.0392	Wnt and Hedgehog signaling pathway
14	0.0013	Dilated cardiomyopathy/tumour migration
12	0.0021	Arrhythmogenic right ventricular cardiomyopathy (ARVC)/Tumour EMT
11	0.0088	Protein digestion and absorption/ECM components
16	0.0058	Cell adhesion molecules (CAMs)
12	0.0061	Hematopoetic cell lineage
15	0.0057	Glutamatergic synapse
35	<0.0001	Neuroactive ligand-receptor

(Hyp* = Corrected hypergeometric pValue)

**Table 2:** KEGG analysis for CAIX-ve cells

Genes	Hyp*	Annotations
15	0.0007	Cytokine-cytokine receptor interaction
13	0.0072	MAPK signaling pathway
8	0.0203	Osteoclast differentiation
6	0.0341	ECM-receptor interaction
6	0.0376	TGF-beta signaling pathway

### The CAIX+ve cells are enriched with cells expressing stem cell markers

**Figure 4:**
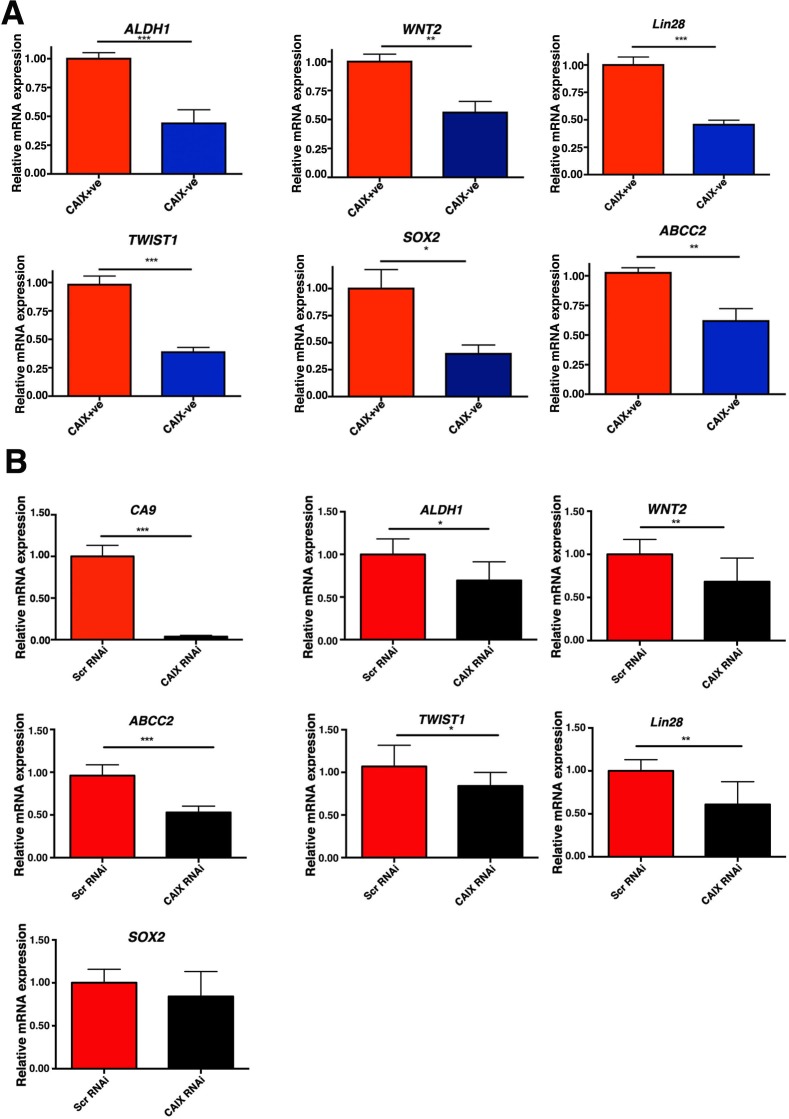
The CAIX+ve subpopulation is enriched with cells expressing stem cell genes **A.** Q-PCR analysis of genes implicated in stem cell maintenance in CAIX+ve and CAIX-ve populations in MCF-7 cells. **B.** Q-PCR analysis of genes implicated in stem cell maintenance in CAIX+ve cells transfected with Scr RNAi or CAIX RNAi. Error bars represent the mean ± SD. ***p < 0.001, **p < 0.01, *p < 0.05, *n* = 3.

Several of the stem cell related genes were validated by Q-PCR and were differentially expressed in the MCF-7 CAIX+ve sorted cells. We found that in CAIX+ve cells expression of ALDH1 (*p* < 0.001, *n* = 3), WNT2 (*p* < 0.01, *n* = 3), TWIST1 (*p* < 0.001, *n* = 3), LIN28 (*p* < 0.001, *n* = 3), ABCC2 (*p* < 0.01, *n* = 3), IGF1 (*p* < 0.001, *n* = 3), SOX2 (*p* < 0.05, *n* = 3), was significantly upregulated (Figure 4A). CAIX silencing in the CAIX+ve cell line led to reduced gene expression of *ALDH1* (*p* < 0.05, *n* = 3), *TWIST1* (*p* < 0.05, *n* = 3), and *ABCC2* (*p* < 0.001, *n* = 3), *WNT2* (*p* < 0.01, *n* = 3), *LIN28* (*p* < 0.01, *n* = 3), but it did not effect the expression of *SOX2* (Figure 4B), showing that CAIX is involved as a mechanism as well as a marker. In addition, the protein expression levels of ALDH1 in the CAIX+ve cells sorted from two different cells lines, MCF7 and HCT116 were also higher compared to the CAIX-ve cells (Figure S4B).

### The CAIX+ve cells are enriched for stem cell like properties compared to CAIX-ve cells

**Figure 5:**
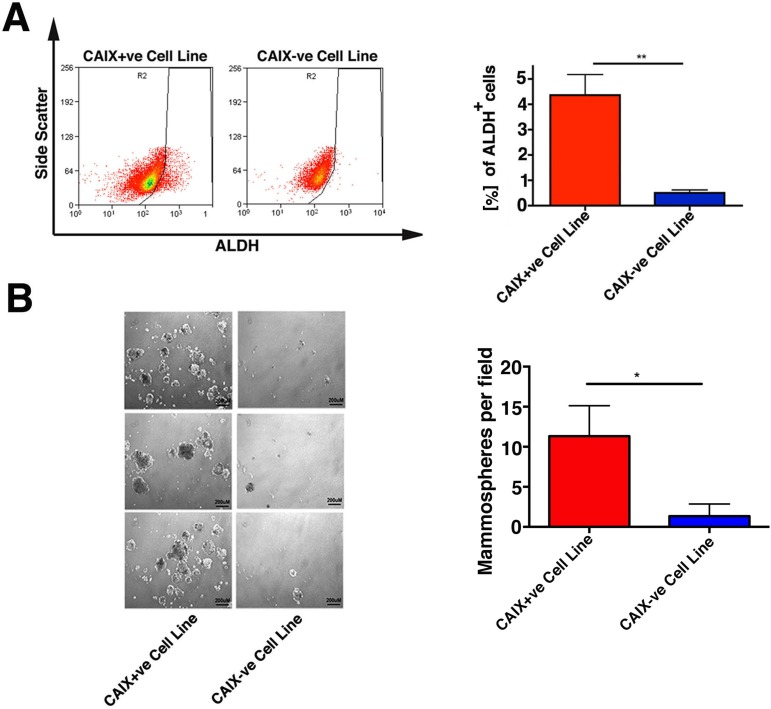
The CAIX+ve subpopulation has functional properties associated with cancer stem cells *in vitro* and *in vivo* **A.** Representative FACS analysis of MCF-7 CAIX+ve and CAIX-ve cell lines for ALDH activity (ALDEFLUOR kit) **B.** The number of mammospheres per microscopic field was determined. Data are shown as the number of mammospheres per field performed in triplicates. Error bars represent the mean ± SD. ***p < 0.001, *p < 0.05, *n* = 3.

We showed that only CAIX+ve cells possess the capacity to drive the original heterogeneous phenotype, a characteristic property of stemness [15, 16]. Importantly, the MCF-7 CAIX+ve cell line had significantly higher ALDH enzymatic activity (*p* < 0.01, *n* = 3) compared to the MCF-7 CAIX-ve cell line (Figure 5A) as analysed by the ALDEFLUOR assay. This is consistent with our hypothesis as high ALDH activity is associated with the self-renewal capacity and ability to recapitulate the heterogeneity of the parental tumour [17]. Interestingly, basal-like breast cancers, which are associated with stem cell phenotypes and high ALDH1 expression also show high CAIX expression [17, 18]. Moreover, our notion that the CAIX+ve population is enriched with stem-like cells is further supported by the fact that these cell has the capacity to form significantly more second-generation mammospheres compared to the CAIX-ve cell line (Figure 5B, *p* < 0.05, *n* = 3), a property known to be associated with mammary stem/progenitor cells [13]. Interestingly, there is no change in cell viability under normoxic or hypoxic conditions between CAIX+ve and CAIX-ve cells (Figure S5).

### CAIX expression promotes tumour growth in NSG mice

**Figure 6:**
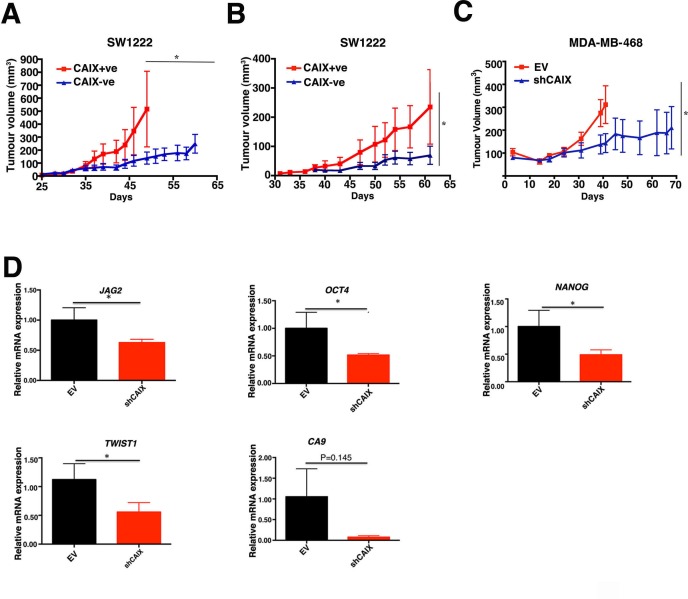
Analysis of CAIX positive and CAIX negative cell lines on ability to promote tumour growth *in vivo* CAIX+ve and CAIX-ve cells derived from SW1222 cells were implanted at 1000 **A.** or 200 cells **B.** and growth monitored over time. **C.** CAIX knockdown reduced the growth rate of MDA-MB-468 xenografts. **D.** Q-PCR analysis of stem cells genes in shMDA-MB-468 and Empty Vector (EV) xenografts. Error bars represent the mean ± SD, *p < 0.05, *n* = 3.

We selected the SW1222 colon cancer cells instead of MCF-7 breast cancer for our *in vivo* studies in order to avoid the potential confounding effects of estrogen supplementation. SW1222 has been previously utilized successfully to model differential stem cell phenotypes in this way previously [10] and has two clear CAIX subpopulations (Figure 1A). These cells differentially express the hypoxic marker CAIX (Figure 1A). Importantly, only the SW1222 CAIX+ve population expresses the CD44^+^CD24^+^ stem cell markers [10] (Figure S6). The SW1222 CAIX+ve cells formed tumours significantly faster (*p* < 0.05, *n* = 5) than the CAIX-ve population, when implanted at 1000 cells (Figure 6A) and with 200 cells (Figure 6B).

Although *in vivo* the role of CAIX in regulating pH may affect the gene expression patterns, *in vivo* analysis of effects of CAIX knockdown was conducted to assess if there was concordance of these *in vitro* observations. To this end, we produced a CAIX knockdown MDA-MB-468 (breast cancer) cell line using stable shRNA. The CAIX knockdown significantly reduced xenograft growth rate (*p* < 0.05, *n* = 5), (Figure 6C). Gene ontology analysis of genes revealed that genes associated with stemness were more highly expressed in the CAIX expressing xenografts of MDA-MB-468 compared to the knockdown (Table S1). Q-PCR analysis confirmed significantly higher expression of stem cell associated gene such as: TWIST1 (*p* < 0.05, *n* = 4), JAG2 (*p* < 0.05, *n* = 4), OCT4 (*p* < 0.05, *n* = 4) and NANOG (*p* < 0.05, *n* = 4) (Figure 6D) in the CAIX expressing xenografts of MDA-MB-468 compared to the knockdown. The genes tested are stem cell marker genes. There may be some differences in the relative expression and dependence on stem cell related genes in different systems i.e MCF-7 (ER positive breast cancer cell line) versus MDA-MB-468 (triple receptor negative breast cancer). In addition to this, previous studies have shown that CAIX is upregulated in basal-like breast tumours [19], which are characterized by their stem like phenotype [20]. Given the above and the fact that MDA-MB-231 and MDA-MB-468 are basal-like breast cancer cell lines, we postulate that the more uniform expression of CAIX in these cell lines is due to the higher proportion of stem like cells in these cell lines (Figure S7).

### Epigenetic regulation of the CA9 genomic locus

**Figure 7:**
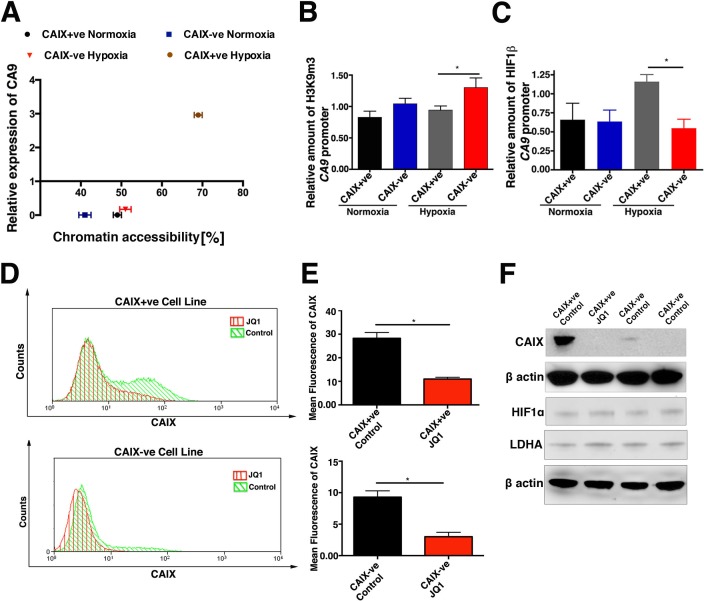
Hypoxic induction of CAIX expression is regulated by chromatin accessibility **A.** Semi-quantitative analysis of chromatin compaction within *CA9* genomic locus in CAIX+ve and CAIX-ve cells under normoxic and hypoxic conditions using EpiQ assay ploted againist relative mRNA expression of *CA9*. **B.** Levels of tri-methyl H3K9 histone at the *CA9* promoter region in CAIX positive and negative cell lines, in normoxia and hypoxia. **C.** Binding of HIF-1β to the *CA9* promoter region in CAIX positive and negative cell lines. **D.** and **E.** JQ1 decreases CAIX expression in CAIX+ve and CAIX-ve cells as analysed by FACS and western blot. HIF1α and LDHA proteins levels **E.** in JQ1 treated CAIX+ve and CAIX-ve cells. Error bars represent the mean ± SD. ***p < 0.001, **p < 0.01, *p < 0.05, *n* = 3.

We hypothesized that differences in chromatin organization were a likely mechanism for CAIX differential regulation. Using the Epiq chromatin analysis kit the chromatin state of a given locus could be interrogated on the basis of its accessibility to nuclease digestion. The MCF-7 CAIX-ve cells displayed a more “closed” chromatin state as shown by the low accessibility to nuclease digestion, which correlates with low mRNA expression of *CA9* (Figure 7A). In contrast, the MCF-7 CAIX+ve cells under hypoxia adopted a more “open” and accessible chromatin state around *CA9* promoter region, which is associated with high expression of *CA9* (Figure 7A). Interestingly both populations under normoxic condition have a compact “silenced” chromatin.

To verify this data we performed chromatin immunoprecipitation (ChIP) on MCF-7 CAIX+ve and CAIX-ve cell lines to evaluate the levels of the tri-methyl H3K9 histone, a well-validated marker of heterochromatin. Under hypoxic conditions the MCF-7 CAIX-ve cell line had significantly (*p* < 0.05, *n* = 3) higher levels of tri-methyl H3K9 histone compared to the MCF-7 CAIX+ve cell line, confirming the nuclease digestion assay (Figure 7B). Given that HIF induces the transcription of *CA9* by binding to HRE in its promoters we hypothesized that this interaction should be reduced in our CAIX-ve cells. A significant (*p* < 0.05, *n* = 3) reduction in binding of HIF1α to the *CA9* promoter of the negative population was identified as determined by ChIP (Figure 7C). In addition, MCF-7 CAIX+ve and CAIX-ve cell lines were treated with A366 a selective inhibitor of the H3K9 methyltransferase G9a/GLP. A366 treatment under hypoxic conditions significantly increased the level of CAIX expression in both MCF-7 CAIX+ve and CAIX-ve cells as analysed by FACS (CAIX+ve *p* < 0.01, *n* = 3; CAIX-ve p = 0.01, *n* = 3)(Figure S8A and B), and western blot (Figure S8C). The drug treatment impacts on chromatin in both populations leading to the change in CAIX expression, which may indicate that the effect of A366 could be indirect. A366 did not affect HIF1α stabilization (Figure S8C) levels of an alternative HIF target gene LDHA.

Further to this MCF-7 CAIX+ve and CAIX-ve cell lines were treated with a selective inhibitor of the bromodomain family JQ1. JQ1 significantly reduced CAIX expression (CAIX+ve *p* < 0.05, *n* = 3; CAIX-ve p = 0.05, *n* = 3) (Figure 7D), but did not affect HIF1α stabilization or LDHA expression (Figure 7E).

### HDAC inhibitors target the cells expressing CAIX

Previous studies have shown that HDAC inhibitors (NaBu and SAHA) induce early differentiation in embryonic stem cells, by reducing the expression of key regulatory stem cell genes such as SOX2, NANOG, and OCT4 [10, 21, 22]. Given the above we tested the effect of NaBu treatment on CAIX expression. We observed that NaBu treatment caused a significant downregulation of CAIX (*p* < 0.01, *n* = 3) expression and the expression of the colon cancer stem cell marker CD133 (*p* < 0.05, *n* = 3) (Figure S9).

**Figure 8:**
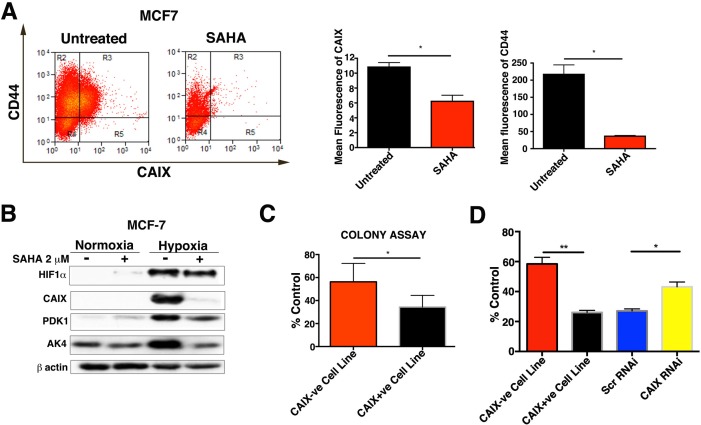
Effects of SAHA treatment on CAIX+ve and CAIX-ve populations **A.** FACS analysis for CAIX and CD44 in hypoxia in SAHA treated and untreated MCF-7. **B.** Protein levels of HIF-1α, CAIX, PDK1 and AK4 in SAHA treated and untreated MCF-7 cells. **C.** Clonogenic survival assay for MCF7 CAIX+ve and CAIX-ve cell lines treated with SAHA. **D.** Effects of SAHA treatment on cell growth of MCF7 CAIX+ve, MCF7 CAIX-ve and MCF7 CAIX knockdown cells. Error bars represent the mean ± SD. **p < 0.01, *p < 0.05, *n* = 3.

In order to verify this result we also investigated the effect of SAHA, in the MCF-7 breast cancer cell line. SAHA treatment significantly downregulated the expression of CAIX (*p* < 0.05, *n* = 3) and the expression of the breast cancer stem cell marker CD44 (*p* < 0.05, *n* = 3) (Figure 8A). The expression of HIF1α was not downregulated upon SAHA treatment, but the two validated HIF and hypoxia regulated genes PDK1 (44% reduction) and AK4 (80%) were, although to a lesser extent than CAIX (95%) (Figure 8B).

Next we investigated whether SAHA induced CAIX downregulation specifically targets MCF-7 CAIX+ve cells by affecting their survival. SAHA treatment significantly (*p* < 0.05, *n* = 3) reduced the number of colonies of the MCF-7 CAIX+ve cell line (Figure 8C) compared to the colonies of the MCF-7 CAIX-ve cell line. Additionally, we evaluated the CAIX expression in untreated and SAHA-treated colonies of the MCF-7 CAIX+ve cell line after the 10d recovery. The expression of CAIX in the SAHA treated colonies was significantly (*p* < 0.05, *n* = 3) reduced compared to the untreated colonies (Figure S10).

To understand if the sensitivity of CAIX+ve cells to SAHA was dependent on the CAIX status we transfected the MCF-7 CAIX+ve population with CAIX RNAi or Scr RNAi and then treated with SAHA for 72 hours under hypoxic conditions. After 72hours SAHA was removed from the medium, then cells were allowed to recover for another 24hours under normoxia and the cell growth was assessed by cell counting. Treatment with SAHA significantly reduced cell growth of the cells transfected with Scr RNAi (*p* < 0.05, *n* = 3) compared to the MCF-7 CAIX knockdown cells (Figure 8D). Moreover, growth of the MCF-7 CAIX+ve cell line (*p* < 0.01,n = 3) was also reduced as compared to the MCF-7 CAIX-ve cell line (Figure 8D). These results suggest that expression of CAIX confers sensitivity to SAHA treatment in the MCF-7 CAIX+ve population. Given the above it is likely that CAIX could be a valuable biomarker in treatment utilizing HDAC inhibitors.

## DISCUSSION

Solid tumours, including breast cancer, are composed of heterogeneous cell populations that interact in complex networks. CAIX heterogeneity is associated with disparities of many stem cell genes. Previous studies have shown that CAIX expression is associated with stem cell niches in normal tissues [11], and plays a crucial role in the invasive potential of breast cancer cells and mammospheres [12, 13]. Here we demonstrate that the CAIX+ve gene signature was significantly enriched for pathways and genes which have been shown to play an important role in the recurrence and maintenance of cancer stem cells [23], [24]. Accordingly, we also show that only CAIX+ve cells possess the capacity to drive the original heterogeneous phenotype, consisting of CAIX-ve and CAIX+ve cells in long-term culture, a characteristic measurement of stemness [15, 16]. Interestingly, our data show that suppression of CAIX *in vitro* and *in vivo* causes a significant downregulation of breast cancer stem cell signatures. This suggest that CAIX does not serve only as a marker for the enrichment of hypoxic cancer stem cells, but its expression induces some stem cell phenotypes. Inhibition of the carbonic anhydrase activity of CAIX has been previously shown to deplete cancer stem cells in tumours [12] *in vivo* suggesting that it is the ability of CAIX to regulate pH that is key for stem cells. It is unlikely that all CAIX+ve cells are stem cells per se or that CAIX-ve cells have no stem cells. Nevertheless, it is intriguing that the CAIX induction program is so strongly associated with genes commonly expressed in stem cells. One possibility is that this creates an extensive microenvironment of regulated pH around the fewer stem cells, a syncytial pH rim, as we showed previously in spheroid models of CAIX expression [25].

We would suggest a term for this population, the hypoxia induced ‘stem cell pH buffer zone’ producing a pH-protected zone enriched for stem cell populations, but having properties conducive to protect them from environmental stress.

Alterations in global levels of specific modifications, such as methylation or acetylation generate an additional layer of epigenetic heterogeneity at the cellular level in tumour tissues. Here we demonstrated that the molecular mechanism underlying the differential expression of CAIX is due to hypoxia induced reorganization of chromatin structure. The CAIX-ve cells have a relatively “closed” and transcriptionally silent CAIX promoter region under hypoxia compared to a transcriptionally active and “open” configuration in the CAIX+ve population. We investigated the relationship between chromatin organization and hypoxic induction of CAIX expression using pharmacological inhibitors of chromatin modulators. Given the higher levels of H3K9 methylation in the CAIX promoter of the negative cells, we considered that inhibition of the H3K9 methylation might induce CAIX in this population. For this reason, we used a selective inhibitor of the H3K9 methyltransferase G9a/GLP (A366). In agreement with our hypothesis, treatment with A366 resulted in induction of CAIX expression in the CAIX-ve population. We also tested the effect of inhibiting a chromatin modification reader, which reads ξ-N-acetylation of lysines, by treatment with JQ1. JQ1 disrupts the binding of BET bromodomains to acetyl lysines in the chromatin, a crucial event for the recruitment of transcription factors necessary for gene activation. In line with this, JQ1 treatment significantly reduced CAIX expression. This highlights the role of epigenetic modification in adaption to hypoxia and also in regulation of heterogeneity of cells within tumours. Drugs targeting chromatin modifiers influence CAIX levels and as such may be used to reduce or target intra-tumour heterogeneity and synergize with anti-VEGF therapy.

In conclusion, using CAIX as a hypoxic marker we have identified for the first time a marked heterogeneity in response to hypoxia within three tumour cell lines, associated with major biological differences. We also identified that NaBu and SAHA treatment lead to elimination of the CAIX+ve population as well as a significant reduction in the expression of the stem cells associated genes CD44 and CD133. Consistent with this, treatment of HT29 and CaCo2 colon cancer cell lines with NaBu also causes a reduction in the expression of CD133 and CD44 [26]. Additionally, it has been shown that HDAC inhibitors induce early differentiation in embryonic stem cells (ES), by reducing the expression of key regulatory stem cell genes such as SOX2, NANOG, and OCT4 [21, 22]. The differential sensitivity to HDAC inhibitors may explain the relatively poor results with this drug class in solid tumours, and suggests that combinations targeting different hypoxic populations should be investigated. The epigenetic mechanism that drives the consistent induction of these two populations implies a key differential hypoxia program that is important for survival of a subpopulation of stem cells in a pH buffer zone. We propose that strategies targeting intra-tumoural heterogeneity using inhibitors of chromatin regulation of heterogeneous expression, including the regulation of CAIX could in combination have significant clinical consequence.

## MATERIALS AND METHODS

### Cell culture

Cells were maintained in a humidified incubator at 5% CO_2_ and 37ºC. 5%. All cell lines were maintained in DMEM supplemented with 10% FBS. Hypoxic incubations (0.1% O_2_, 5% CO_2_,) were performed using an INVIVO_2_ 400 hypoxic workstation (Pro-Lab Diagnostics). Cells were treated with SAHA (Sigma) at 2 μM, NaBu (Sigma) at 5 mM, JQ-1 at 200 nM. A366 and JQ-1 were a gift from Dr Susanne Muller-Knapp (Target Discovery Institute, University of Oxford, UK). Spheroids were generated following the method described in [27].

### Flow cytometry

Flow cytometry was carried out as previously described [28]. Samples were analyzed using a DakoCytomation Cyan machine, or sorted using a MoFlo cell sorter (Beckman Coulter). The bottom 10% of CAIX negative subpopulation and the top 10% of CAIX positive subpopulation were collected for further experiments. The following primary antibodies were used FITC conjugated CAIX (R&D systems), APC conjugated CD133 (Miltenyi Biotec), APC conjugated CD44 antibody (BD Biosciences) and PE conjugated CD24 antibody (BD Biosciences).

### Chromatin analysis

The quantitative assessment of chromatin states of CAIX+ve and CAIX-ve cells was performed using the EpiQ Chromatin analysis kit according to manufacturer's instructions (BioRad). Briefly CAIX+ve cell line and CAIX-ve cell line were plated in 48 well culture plate to 80-95% confluent and exposed for 72hrs in 0.1% O_2_ or they were maintained under normoxic conditions. Three wells remained undigested and the other 3 wells were digested with DNase nuclease I for 1 hour. Following extraction and purification of the genomic DNA. The nuclease sensitivity was assessed by qPCR (with primers as shown in Table S6). The data were analysed using EpiQ Chromatin Kit Data Analysis Tool (www.bio-rad.com/epiq) The GAPDH region was used as a positive reference gene region for an open chromatin.

### Chromatin immunoprecipitation assay

Immunoprecipitation assays for HIF-1α were performed using the Upstate EZ ChIP Kit Reagents and chromatin immunoprecipitation (ChIP) assay for trimethylated H3K9 was done using the EpiQuik Tri-Methyl-Histone H3K9 ChIP Kit (Epigentek, Brooklyn, NY) according to manufacturer's instructions.

### Aldefluor assay

To detect ALDH activity in cells, we used the ALDEFLUOR kit from Stem Cell Technologies, according to manufacturer's instructions.

### Generation of mammospheres

MCF-7 cells were detached, washed and plated in a 6 well low attachment plates (Corning), filled with 3mL MammoCult Basal Medium and supplemented with MammoCult proliferation Supplements, 4 μg/mL Heparin, 0.48 μg/mL Hydrocortisol and penicillin (100 U/mL), streptomycin (100mg/mL). MCF-7 mammospheres were started forming after day 4-6 and were processed at day 10. Secondary generation mammospheres were generated by incubating the primary mammospheres with 1 X Trypsin EDTA solution (PAA Laboratories) for 2-3 min and resuspended in the above medium and plated again in the low attachment plates. After 7 days the capacity of cells to generate secondary mammospheres were tested by visualizing the cells with the inverted light microscope (Axiovert).

### Production of stable CAIX knockdown MDA-MB-468 cells

To knockdown *CA9* in MDA-MB-468, the HuSH-29 shRNA targeting *CA9* (TR314250) and empty vector (R20003) were purchased from Origene. The shCA9 sequence was cloned into pcDNA3.1. Cell lines were transfected with FuGENE 6 (Roche) according to manufacturer's instructions. Cell line pools were grown under selective pressure [0.4 mg/mL G418 (Invitrogen)] until no mock-transfected cells remained.

### Immunoblotting

Cell lysates were separated on 10% SDS-PAGE and transferred to polyvinylidene difluoride membrane. Primary antibodies were used at 1:1,000 unless otherwise stated. The following antibodies were used, mouse anti-HIF1α, (BD Transduction Laboratories, USA), mouse anti-CAIX (Gift form J. Pastrorek, Institute of Virology, Slovak Republic), goat anti-LDHA (Santa-Cruz), goat anti-PDK1 (Santa-Cruz), goat anti-AK4 (Santa-Cruz), mouse anti-ALDH (BD Biosciences), mouse anti-β actin (Sigma). Blots were quantified with image analysis in ImageJ.

### Real-time PCR (Q-PCR)

RNA extraction and the quantitative PCR protocol have been described previously (33). The sequences of the primers used are showing in Table S2.

### RNA-sequencing and gene expression array analysis

RNA was extracted as described above. The RNA-seq was performed according to the sample preparation guide (Illumina, TruSeq RNA). TopHat [29] was used to align RNA-Seq reads to the human genome build 37.1. Bedtools [30] was used to summarize the number of reads per exon per transcript. Threshold for transcript presence was set to 15 reads. Samples were then normalized using the total number of mapped reads. The logarithm base 2 of the ratio of counts between conditions was estimated and a filter was applied to select genes with an absolute logarithm greater than 1; this is equivalent to one fold change up- or down-regulation between conditions.

Enrichment for KEGG pathways (http://www.genome.jp/kegg/) was performed using Genecodis 2.0 [31, 32]; Ensembl IDs (GRCh37 assembly) were used as reference, hypergeometric test was used and false discovery rate threshold was set to 0.05.

For Illumina expression. Briefly, RNA concentration was normalized to 50ng/ul of which 11ul used to produce biotin-labelled complementary RNA (cRNA) using Illumina TotalPrep-96 RNA amplification kit from Ambion #4393543. 750ng of biotin-labelled cRNA from each sample was hybridized according to Illumina whole-genome gene expression direct hybridization assay from Illumina #11286340 to high-density Illumina Human oligonucleotide arrays Human HT-12_V4_0_R1_15002873_B; designed to detect 47,231 transcripts. The Fluorescence emissions were quantitatively detected using iScanner and data were extracted using BeadStudio v20111.1 Software (Illumina Inc) which were imported to GeneSpring GX 12.1 (Agilent Technologies, Inc., Santa Clara, CA) ; normalized with Shift to 75 percentile and baseline transformed to median of all samples to identify significantly differentially expressed genes with FDR (Benjamini-Hochberg) corrected *P*-value cut off at < 0.01. Functional and pathway analysis carried out on differentially expressed genes to identify statistically over-represented ontologies in the list using Database for Annotation, Visualization and Integrated Discovery (DAVID; Huang da et al., 2009b; Huang da et al., 2009a).

### Xenograft studies

Mice were housed at BMS, University of Oxford, UK, and procedures were carried out under a Home Office licence. SW1222 cells were exposed in hypoxia for 72h and then were sorted in CAIX+ve and CAIX-ve population. 1000 CAIX+ve, CAIX-ve and 200 CAIX+ve, CAIX-ve cells were inoculated in six to seven-week old male NSG mice subcutaneously in the lower flank with 100μL Matrigel (BD Bioscience). MDA-MB-468 EV and shCA9 cells were inoculated in six to seven-week old female BALB/c NuNu mice subcutaneously in the lower flank with 1×10^7^ with 50μL Matrigel (BD Bioscience). Tumour growth was monitored three times per week measuring the length (L), width (W) and height (H) of each tumour using calipers. Volumes were calculated from the formula 1/6 x π x L x W x H. When tumours reached 1.44 cm^3^ the mice were sacrificed by cervical dislocation. Ninety minutes prior to sacrifice, mice were injected intravenously with 2 mg of pimonidazole (Hypoxyprobe-1; Chemicon International, USA) as described previously [33].

### Gene silencing by RNA interference

Transfections of siRNA duplexes targeting CAIX or a scramble control (scr) (ON-TARGETplus SMARTpool) at a final concentration of 20 nM, were performed in Optimem (Invitrogen), using Oligofectamine reagent (Invitrogen). The sequences of siRNA used to target CAIX were 118898, 9567 and 9473 (Invitrogen).

### Clonogenic assay

CAIX+ve and CAIX-ve cells were seeded at a density of 1000 cells per 6 cm dish in triplicates and treated with 2 μM SAHA for 72h under hypoxic conditions, untreated control cells were also included in the experiment. After 72h the dishes incubated in hypoxia were returned to normoxia for 10 days and allowed to form colonies. Cell colonies were fixed and stained with Methylene blue for 1 hour. Visible colonies were counted using a ColCount automated colony counter (Optronix).

### Statistical analyses

Statistical analysis and graphs were performed using GraphPad Prism® v4.0 software (GraphPad). Statistics were carried out using non-paired Student's *t*-tests and significance is represented by *p < 0.05, **p < 0.01, and ***p < 0.001. Error bars represent mean ± Standard Deviation (SD).

## SUPPLEMENTARY MATERIAL FIGURES AND TABLES


