# Supplementary material for: Carbonic anhydrase IX induction defines a heterogeneous cancer cell response to hypoxia and mediates stem cell-like properties and sensitivity to HDAC inhibition
Source: Oncotarget. 2015 Jul 22;6(23):19413–27. doi: 10.18632/oncotarget.4989 (PMC4637295; doi:10.18632/oncotarget.4989)
Supplement: Supplementary file 1 [file oncotarget-06-19413-s001.pdf]

Carbonic anhydrase IX induction defines a heterogeneous cancer cell response to hypoxia and mediates stem cell-like properties and sensitivity to HDAC inhibition

Supplementary Material

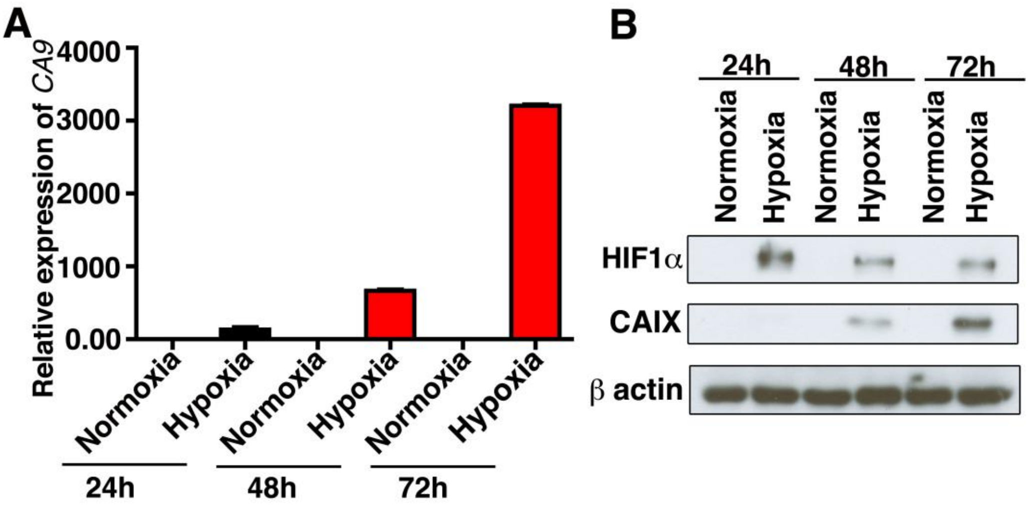

Figure S1: Expression of CAIX in MCF7 breast cancer cell line. MCF7 breast cancer cell line shows no expression of CAIX under normal growth condition and an upregulation of CAIX in hypoxia, with higher levels of expression at 72h at both mRNA (A) and protein level (B). Error bars represent SD.

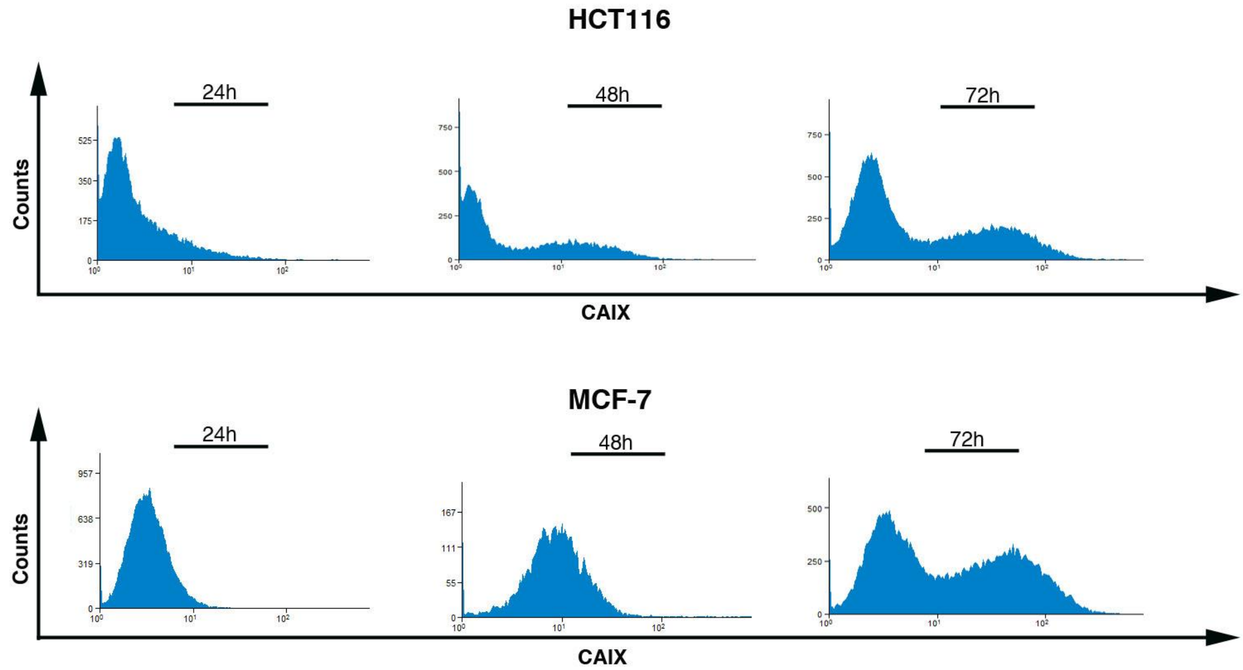

Figure S2: Time points for CAIX expression in colon cancer cell line HCT166 and breast cancer cell line MCF-7. Representative FACS analysis of HCT166 and MCF-7 cell lines for CAIX expression at 24hours, 48 hours and 72hours under hypoxic conditions.

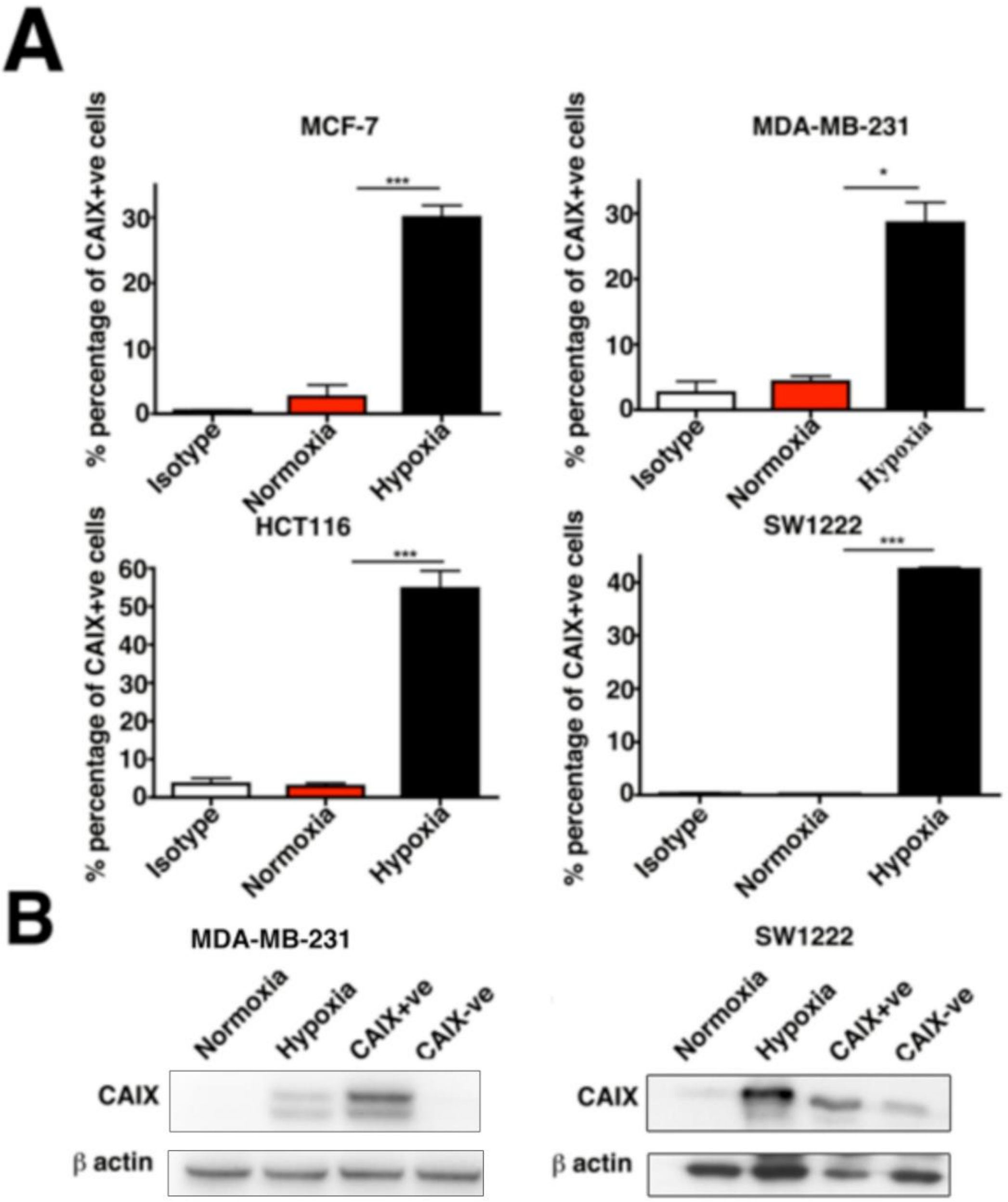

Figure S3: The percentage of CAIX+ve cells in breast and colorectal cancer cell lines. (A) Percentage of CAIX+ve cells under hypoxia in MCF-7, MDA-MB-231, HCT116 and SW1222 cell lines. (B) The expression level of CAIX of the sorted MDA-MB-231 and SW1222 cells was confirmed at protein level. Error bars represent the mean ± SD.

A

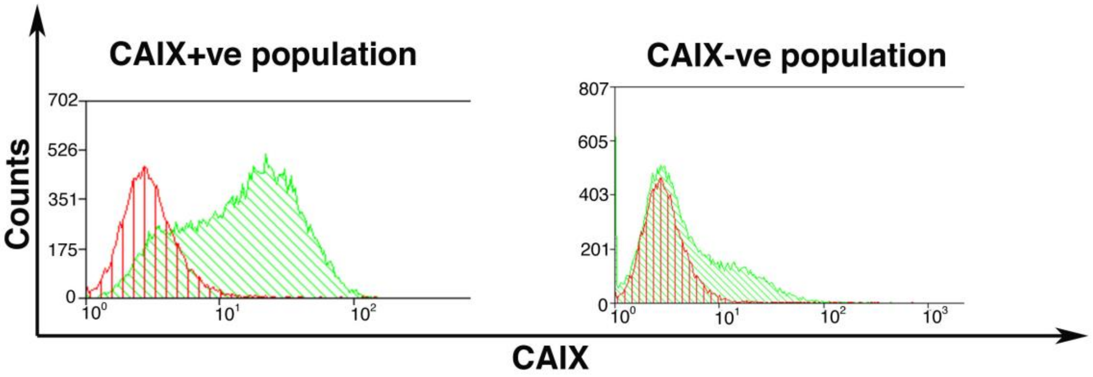

Mean Fluorescence	MCF7 CAIX+ve	MCF7 CAIX-ve	MCF7 Normoxia
Anti-CAIX	20.75	7.15	5.48

B

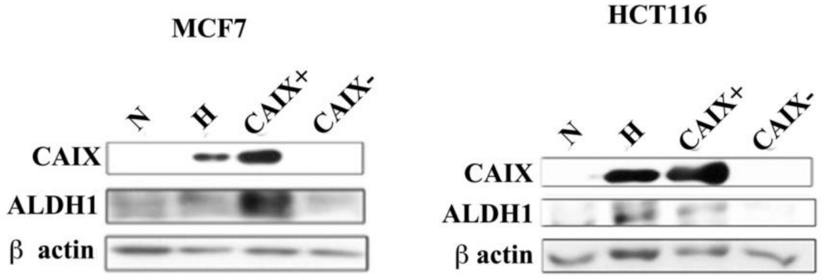

Figure S4: **Expression profile of CAIX+ve and CAIX-ve sorted cells cultured for 3 weeks.** (A) The CAIX+ve population had recapitulated the original unsorted phenotype, generating both populations of CAIX+ve and CAIX-ve cells. Red peaks represent cells in normoxia. Green peaks represent cells in hypoxia. (B) Protein levels of ALDH1 in MCF-7 and HCT116 cell lines.

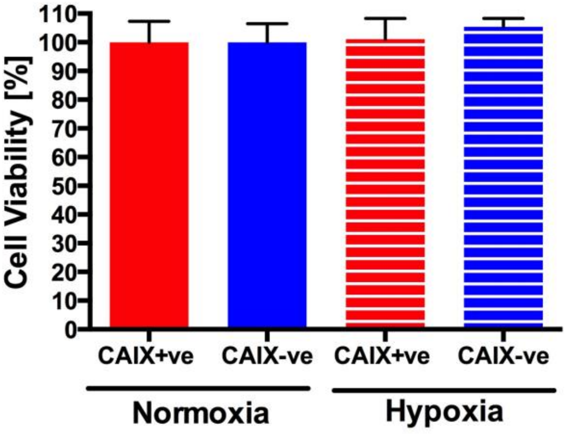

Figure S5: Cell viability analysis of CAIX+ve and CAIX-ve cells. Measurement of cell viability after culture of the CAIX+ve and CAIX-ve cells in normoxia and hypoxia for 72 hours. There is no difference in cell viability between CAIX+ve and CAIX–ve MCF7 cells over a 72 hour time course in normoxia and hypoxia.

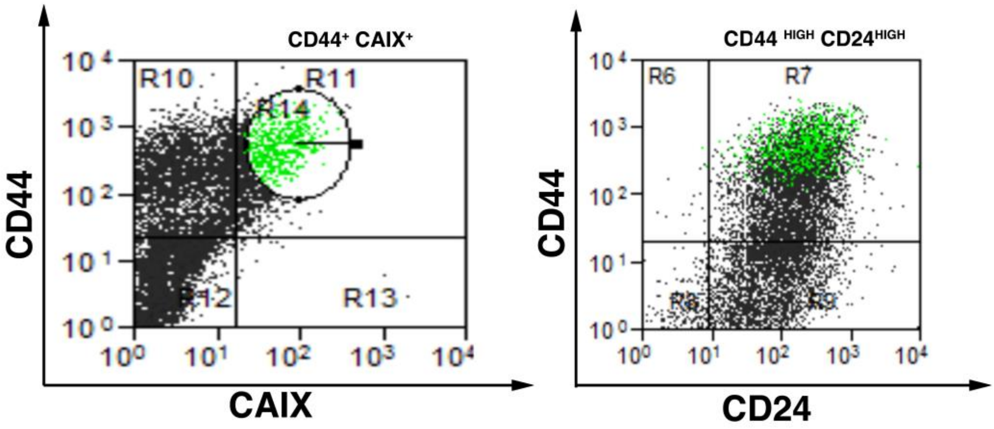

Figure S6: CAIX expression co-localizes with the expression of stem cell markers. CAIX+ve SW1222 colon cancer cells express the stem cells markers CD44^{+HIGH}CD24^{+HIGH}.

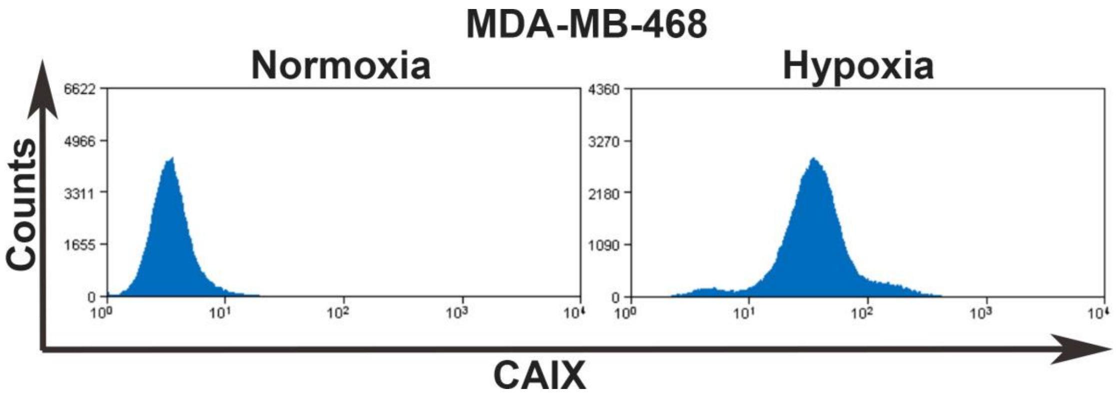

Figure S7: Expression profile of MDA-MB-468 breast cancer cell line. Representative FACS analysis of MDA-MB-468 cell line for CAIX expression under normoxic and hypoxic conditions.

Table S1: Processes and Genes Downregulated in CAIX Knockdown Xenografts

Genes	p-value	Annotations	Gene Identifier
9	0.0125394	Development protien	ILMN_1724480, ILMN_1664861, ILMN_1798496, ILMN_1677429, ILMN_2351272, ILMN_2314007, ILMN_1764729, ILMN_1790338, ILMN_2316236
5	0.00951824	cell:cell junction	ILMN_2188521, ILMN_1663575, ILMN_1785646, ILMN_1769388, ILMN_2399503
4	0.01068787	regulation of cell migration	ILMN_2396875, ILMN_1780825, ILMN_1746085, ILMN_2342695, ILMN_1764729
8	0.01758018	regulation of proliferation	ILMN_1724480, ILMN_2396875, ILMN_1785646, ILMN_1746085, ILMN_2342695, ILMN_1764729, ILMN_2219002, ILMN_1790338, ILMN_2404688
5	0.01282055	regulation of growth	ILMN_2396875, ILMN_1802706, ILMN_1756469, ILMN_1746085, ILMN_1787186, ILMN_2316236
4	0.01310573	Homebox genes	ILMN_1798496, ILMN_1657606, ILMN_1790338, ILMN_2316236
8	0.04289153	cell projection organisation	ILMN_2060413, ILMN_1669123, ILMN_1713749, ILMN_2238302, ILMN_1743199, ILMN_2339955, ILMN_1716687, ILMN_1687301

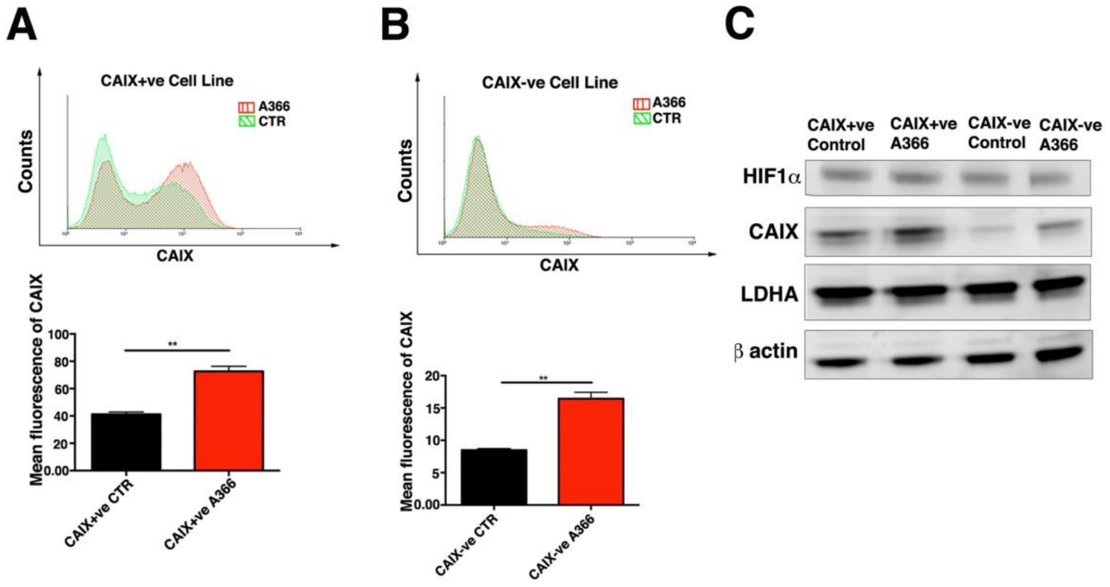

Figure S8: Effect of A366 treatment in CAIX expression. A366 increases CAIX expression in MCF-7 CAIX+ve and CAIX-ve cells as analysed by FACS (A and B) western blot under hypoxic conditions (C). HIF1α and LDHA and protein levels in A366 treated MCF-7 CAIX+ve and CAIX-ve cells.

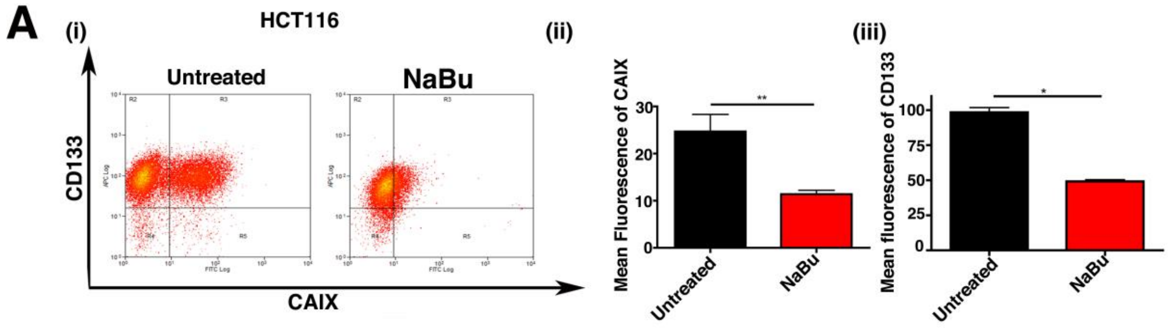

Figure S9: Treatment of unsorted HCT116 cells with sodium butyrate. (A) Representative time point of FACS analysis for CAIX and CD133 expression under hypoxia in NaBu treated and untreated HCT116 cells. (i) Mean fluorescence of CAIX and CD133 (ii) in untreated cells and NaBu treated cells (iii). Error bars represent the mean ± SD.**p<0.01, *p<0.05, n=3.

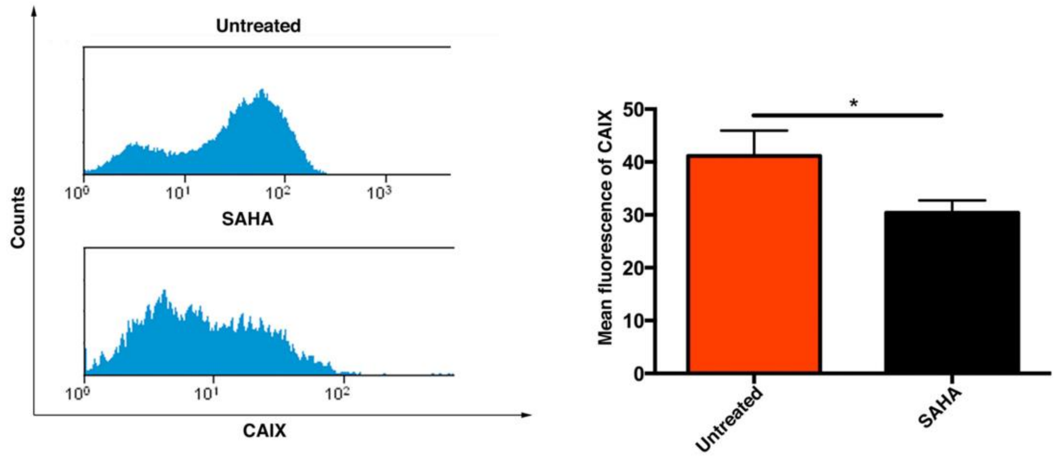

Figure S10: **Effects of SAHA treatment on colony formation of MCF-7 CAIX+ve cell line.** Representative histograms from FACS-based analysis of CAIX expression in untreated and SAHA treated CAIX+ve colonies after 10d recovery. Error bars represent the mean ± SD. *p<0.05, n=3

Table S2: Gene Primers

Gene Name	Forward Primer	Reverse Primer
CA9	*CTTGGAAGAAATCGCTGAGG;*	*TGGAAGTAGCGGCTGAAGTC*
LDHA	*CAGAAGGCTCGAGAAGGTATATTGCTGTTGACAGTGAGCG*	*CCTAAAGTAGCCCCTTGAATTCCGAGGCAGTAGGCA*
PDK1	*CTGGGTAATGAGGATTTGACTGT*	*AAGTCTGTCAATTTTCCTCAAAGG*
ALDH1	*CGGGAAAAGCAATCTGAAGAGGG*	*GATGCGGCTATACAACACTGGC*
WNT2	*AGGATGCCAGAGCCCTGATGAA*	*AGCCAGCATGTCCTGAGAGTAC*
TWIST1	*GCCAGGTACATCGACTTCCTCT*	*TCCATCCTCCAGACCGAGAAGG*
LIN28	*CCAGTGGATGTCTTTGTGCACC*	*GTGACACGGATGGATTCCAGA;*
ABCC2	*GCCAACTTGTGGCTGTGATAGG*	*ATCCAGGACTGCTGTGGGACAT*
IGF1	*CTCTTCAGTTCGTGTGTGGAGAC*	*CAGCCTCCTTAGATCACAGCTC*
SOX2	*GCTACAGCATGATGCAGGACCA*	*TCTGCGAGCTGGTCATGGAGTT*
β-actin	*CCCAGCACAATGAAGATCAA*	*CGATCCACACGGAGTACTTG*
CA9 HREs	GTCCATGGCCCCGATAACCTTCTG	GGGGCAACCTCTGGGGATGGAC
GAPDH	CGCACGTAGCTCAGGCCTCAAGACC	GGCTGACTGTCGAACAGGAGGAGCA
RHO	GGCTGACTGTCGAACAGGAGGAGCA	CCTTGGCCCCTCTTAGAAGCCAATT
